# Heterogeneity in threat extinction learning: substantive and methodological considerations for identifying individual difference in response to stress

**DOI:** 10.3389/fnbeh.2013.00055

**Published:** 2013-05-29

**Authors:** Isaac R. Galatzer-Levy, George A. Bonanno, David E. A. Bush, Joseph E. LeDoux

**Affiliations:** ^1^Department of Psychiatry, New York University School of MedicineNew York, NY, USA; ^2^Department of Counseling and Clinical Psychology, Columbia UniversityNew York, NY, USA; ^3^Center for Neural Science, New York UniversityNew York, NY, USA

**Keywords:** fear extinction learning, heterogeneity, latent growth modeling, PTSD

## Abstract

Pavlovian threat (fear) conditioning (PTC) is an experimental paradigm that couples innate aversive stimuli with neutral cues to elicit learned defensive behavior in response to the neutral cue. PTC is commonly used as a translational model to study neurobiological and behavioral aspects of fear and anxiety disorders including Posttraumatic Stress Disorder (PTSD). Though PTSD is a complex multi-faceted construct that cannot be fully captured in animals PTC is a conceptually valid model for studying the development and maintenance of learned threat responses. Thus, it can inform the understanding of PTSD symptomatology. However, there are significant individual differences in posttraumatic stress that are not as of yet accounted for in studies of PTC. Individuals exposed to danger have been shown to follow distinct patterns: some adapt rapidly and completely (resilience) others adapt slowly (recovery) and others failure to adapt (chronic stress response). Identifying similar behavioral outcomes in PTC increases the translatability of this model. In this report we present a flexible methodology for identifying individual differences in PTC by modeling latent subpopulations or classes characterized by defensive behavior during training. We provide evidence from a reanalysis of previously examined PTC learning and extinction data in rats to demonstrate the effectiveness of this methodology in identifying outcomes analogous to those observed in humans exposed to threat. By utilizing Latent Class Growth Analysis (LCGA) to test for heterogeneity in freezing behavior during threat conditioning and extinction learning in adult male outbred rats (*n* = 58) three outcomes were identified: rapid extinction (57.3%), slow extinction (32.3%), and failure to extinguish (10.3%) indicating that heterogeneity analogous to that in naturalistic human studies is present in experimental animal studies strengthening their translatability in understanding stress responses in humans.

## Introduction

Behavioral responses to environmental cues that signal impending danger are biologically programmed, highly conserved functional adaptations that facilitate rapid threat response (LeDoux, 1996; Rosen and Schulkin, 1998). Included are freezing, escape, potentiated startle, and increased vigilance. The mechanisms underlying the acquisition and control of threat responses have been effectively studied in laboratory experiments in rodents using a procedure called Pavlovian fear conditioning, or more accurately, Pavlovian *threat* conditioning (PTC) (LeDoux, 1996, 2000; Fanselow and Poulos, 2005; Maren, 2005). In PTC an emotionally neutral conditioned stimulus (CS), such as a tone, is paired with an aversive unconditioned stimulus, typically footshock, resulting in the CS acquiring the capacity to elicit behavioral defense responses (freezing) and corresponding changes in autonomic nervous system activity and stress hormones release. Repeated presentations of the CS without the aversive stimulus typically leads to threat (fear) extinction learning whereby the defense responses are weakened as a result of the loss of the predictive value of the CS (Morgan et al., 1993; Myers and Davis, 2002; Quirk and Mueller, 2007; Sotres-Bayon et al., 2008).

Extinction learning in particular, may be relevant to PTSD, as this disorder has been conceptualized as a failure to extinguish an elevated physiological threat response following significant danger or harm (Bryant, 2003; Milad et al., 2006; Yehuda and LeDoux, 2007; Jovanovic and Norrholm, 2011; Norrholm et al., 2011). Research has capitalized on this conceptualization to make advances in understanding mechanisms of and treatments for PTSD. Neurobiological features of extinction learning in both rodents and humans have been extensively studied to better understand the development and maintenance of posttraumatic stress. Further, behavioral approaches meant to encourage extinction learning including exposure therapy and novel treatments involving the interference with memory reconsolidation are key features of treatments for PTSD and other anxiety disorders (Monfils et al., 2009; Schiller et al., 2009; Milad and Quirk, 2012).

Despite the relevance of PTC paradigms to the study of PTSD, there are glaring differences between animal and human studies that hamper their translatability. PTSD develops in a small subset of individuals who have an extreme persistent reaction after exposure to stress. Longitudinal studies have shown that there is clinically relevant heterogeneity in the course of PTSD symptoms over time (Bonanno, 2004, 2005, 2006; Bonanno et al., 2011) with distinct populations, include rapid adaptation with only transient symptoms (*resilience*), slow remission (*recovery*), and failure to remit (*chronic PTSD*) (Deroon-Cassini et al., 2010; Galatzer-Levy et al., 2011a; Bonanno et al., 2012b). By contrast, models of PTC have primarily relied on the analysis of central tendencies which ignore heterogeneity (Sotres-Bayon et al., 2008) and assume that the group average represents individual behavior overall (Judd et al., 2009). Nonetheless, heterogeneity has been observed in stress-related behavior in rats exposed to identical experimental conditions casting doubt on the value of central tendencies for studying PTC (Cavigelli and McClintock, 2003; Cavigelli et al., 2006; Bush et al., 2007).

Heterogeneity in animal models is has gained attention recently (Bush et al., 2007; Duvarci et al., 2009) and is theorized to have important repercussions for translational models of stress response in humans (Bush et al., 2007; McEwen et al., 2012). However, there has been very little discussion of methodological considerations regarding identification or examination of heterogeneity in animal models of PTC. Addressing this gap is important for fleshing out the theoretical connection between learned defensive responses and genetic polymorphisms, epigenetics, and key molecular circuits that may differentiate clinically relevant outcomes such as posttraumatic stress and resilience (Martin et al., 2010; Holmes and Singewald, 2013).

Attempts have been made to identify heterogeneous behavioral phenotypes among animals exposed to threat by first determining the mean response and then identifying populations of animals whose behavior deviates from that mean by one or two standard deviations (Bush et al., 2007; Krishnan et al., 2007). However, in animals, highly non-normal distributions have been observed in defensive behaviors (Cavigelli and McClintock, 2003; Bush et al., 2007; Sotres-Bayon et al., 2008; McEwen et al., 2012), making both the mean and standard deviation considerably less informative. Similarly non-normal distributions in symptoms of PTSD and general distress following exposure in humans have been observed and found to be indicative of behavioral heterogeneity. Heterogeneous patterns have been identified using modeling techniques that reveal latent sub-populations, or “classes,” within finite distributions in longitudinal behavioral measures (Galatzer-Levy et al., 2011a, 2012, 2013).

A relatively new set of modeling techniques, termed Latent Growth Modeling (LGM), is specifically suited to identify parameters of heterogeneous trajectories in longitudinal data by identifying such finite distributions (Muthen, 2004). This approach provides a method to empirically examine heterogeneous latent classes that are distinguished by their pattern of change over time. Importantly, LGM provides statistical methods for determining the number of classes that best fit the data and a framework for statistically testing hypotheses related to that heterogeneity (Del Boca et al., 2004).

The LGM approach has been utilized to identify and explore heterogeneity in situations where it may not be parsimonious to assume one common behavioral pattern, including drinking behavior among college students (Greenbaum et al., 2005), childhood aggression (Schaeffer et al., 2003), and developmental learning trajectories (Boscardin et al., 2008). More recently, an LGM approach has been utilized to identify common patterns of symptom and distress response to potentially traumatic life events, including trajectories of depression and anxiety symptoms following traumatic spinal cord injury (Bonanno et al., 2012a) and distress and posttraumatic stress symptoms following trauma exposure (Galatzer-Levy et al., 2011c, 2013; Bonanno et al., 2012b; Galatzer-Levy and Bonanno, 2013).

Given that extinction learning following PTC is widely considered a strong translational model of behavioral responses to traumatic stress (Milad et al., 2009; Milad and Quirk, 2012), we hypothesize that similar trajectories of behavioral response would be identifiable in rodent extinction as in humans responding to traumatic stress. In the current investigation, we therefore tested the hypothesis that freezing behavior during extinction of PTC in rodents would be characterized better by discrete heterogeneous trajectories of growth in freezing behavior than by measures of central tendency. Specifically, we hypothesized that distinct patterns of rapid extinction, slow extinction, and failure to extinguish threat learning would be identifiable, consistent with observations of the heterogeneous course of stress pathology and resilience in humans. To achieve this aim, we reanalyzed data used in a previous study of individual differences in rat freezing behavior in response to this learning paradigm to examine individual differences patterns using LGM-based modeling techniques (Bush et al., 2007).

## Materials and methods

### Participants

The current investigation examined *n* = 58 adult male outbred Sprague-Dawley strain rats previously reported on Bush et al. (2007). Seven rodents who had been excluded as outliers in the previous analysis were included in this study. The rats weighed 275–325 g upon arrival (Hilltop Lab Animals, Inc., Scottsdale, PA) and were individually housed in transparent plastic Nalgene cages. The animals were maintained throughout the experiment on a 12/12-h light/dark cycle in a temperature- and humidity-controlled environment with food and water available *ad-libitum*. All procedures were in accordance with the National Institute of Health *Guide for the Care and Use of Experimental Animals* and were approved by the New York University Animal Care and Use Committee. Data from control rats across previous fear conditioning and extinction studies were culled for the current analysis. This data is from vehicle control rats that were exposed to identical behavioral procedures.

Habituation, defense conditioning, testing, and extinction took place in one of four identical chambers constructed of aluminum and Plexiglas walls and metal stainless steel rod flooring attached to a shock generator. The chambers were lit by a single house light except during the dark cycle. Each chamber was enclosed in a sound-isolation cubicle with an infrared digital camera mounted on top of each chamber used to record behavior. An overhead 24-cell, three dimensional infrared activity sensors continuously monitored all movement in the chamber and data was recorded on a computer equipped with Coulbourn Instruments LabLinc Habitest Universal Linc System at temporal resolution of 20 ms. This computer also controlled the stimulus presentation with Graphic State 2 software (Coulbourn Instruments). Chambers were thoroughly cleaned between sessions.

#### Measures and procedures

Freezing behavior was used as a behavioral measure of defensive response to the CS. Freezing in this context is defined as the cessation of all movement with the exception of respiration-related movement and non-awake or rest body posture (McAllister and McAllister, 1971). An automated scoring method that collects activity/inactivity data with the overhead infrared activity monitor was used to measure freezing behavior. Data was converted to quantitative freezing values using custom MATLAB® (The Math Works, Inc., Natick, MA) code, where freezing was defined as continuous inactivity lasting at least 2 s. Videotaped behavior was used to monitor behavior during extinction to ensure that resting/sleeping was not scored as freezing.

Conditioning was conducted in groups of four rats at a time, with all rats first exposed to five habituation trials (CS-alone presentation). The following day, rats were given auditory conditioning, beginning 4 min after placement in the chamber. Conditioning consisted of seven tone presentations (30 s, 5 kHz, 80 dB SPL), each co-terminating with a footshock (1 s, 0.7 mA). The mean inter-trial interval was 4 min in the 2–6-min range throughout habitation consistent with other studies of auditory fear conditioning (Milad and Quirk, 2002). Rats were returned to their home cages in the colony room immediately following conditioning. Extinction training commenced one day following conditioning and consisted of 20 CS-alone presentations given in the conditioning context.

### Data analysis

Latent Class Growth Analysis (LCGA) was employed using Mplus 6.12 (Muthen and Muthen, 2006) to identify heterogeneous trajectories by testing for discrete mixture distributions of threat conditioning and extinction. LCGA is an extension of LGM that utilizes fixed effects. Fixed effects were employed to reduce the number of parameters being modeled because of the relatively small sample size. We utilized a piecewise model. Piecewise models allow one to model separate trajectories for the same participants. In this context, a piecewise model was used because we wanted to examine a trajectory for threat conditioning and a separate trajectory for threat extinction. The threat conditioning piece included seven time points and the extinction piece covered 20 time points. The two trajectories were connected by a single intercept placed at the first extinction trial. We compared competing models with linear slopes only for conditioning and extinction, as well as linear and quadratic patterns for a progressive number of classes. The best fit in terms of linear or linear + quadratic was assessed, as well as the best fitting number of classes based on the information criteria [Bayesian (BIC), sample-size adjusted Bayesian (SSBIC), Aikaike Information Criterion (AIC)], and the Bootstrap Likelihood Ratio Test (BLRT), along with parsimony and interpretability consistent with recommendations from the literature (Nylund et al., 2007). Models were compared and the best model was selected based on lower values for the criterion indices, and a significant *p*-value for the BLRT. We also examined entropy to assess the likelihood that individual rats were conforming to the modeled trajectories.

## Results

**Figure 1:**
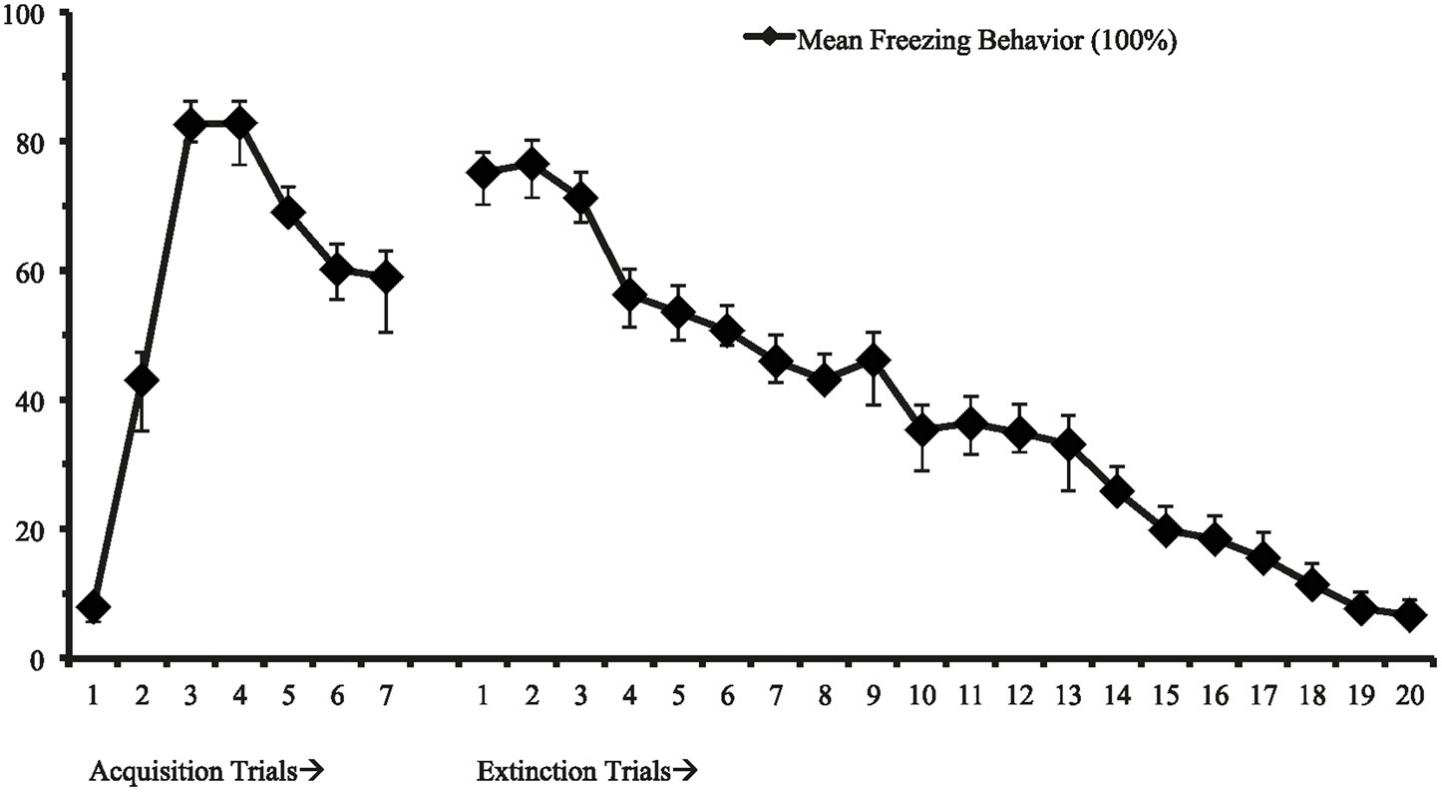
**Mean freezing behavior for 7 fear conditioning and 20 fear extinction trails (*n* = 58)**.

**Figure 2:**
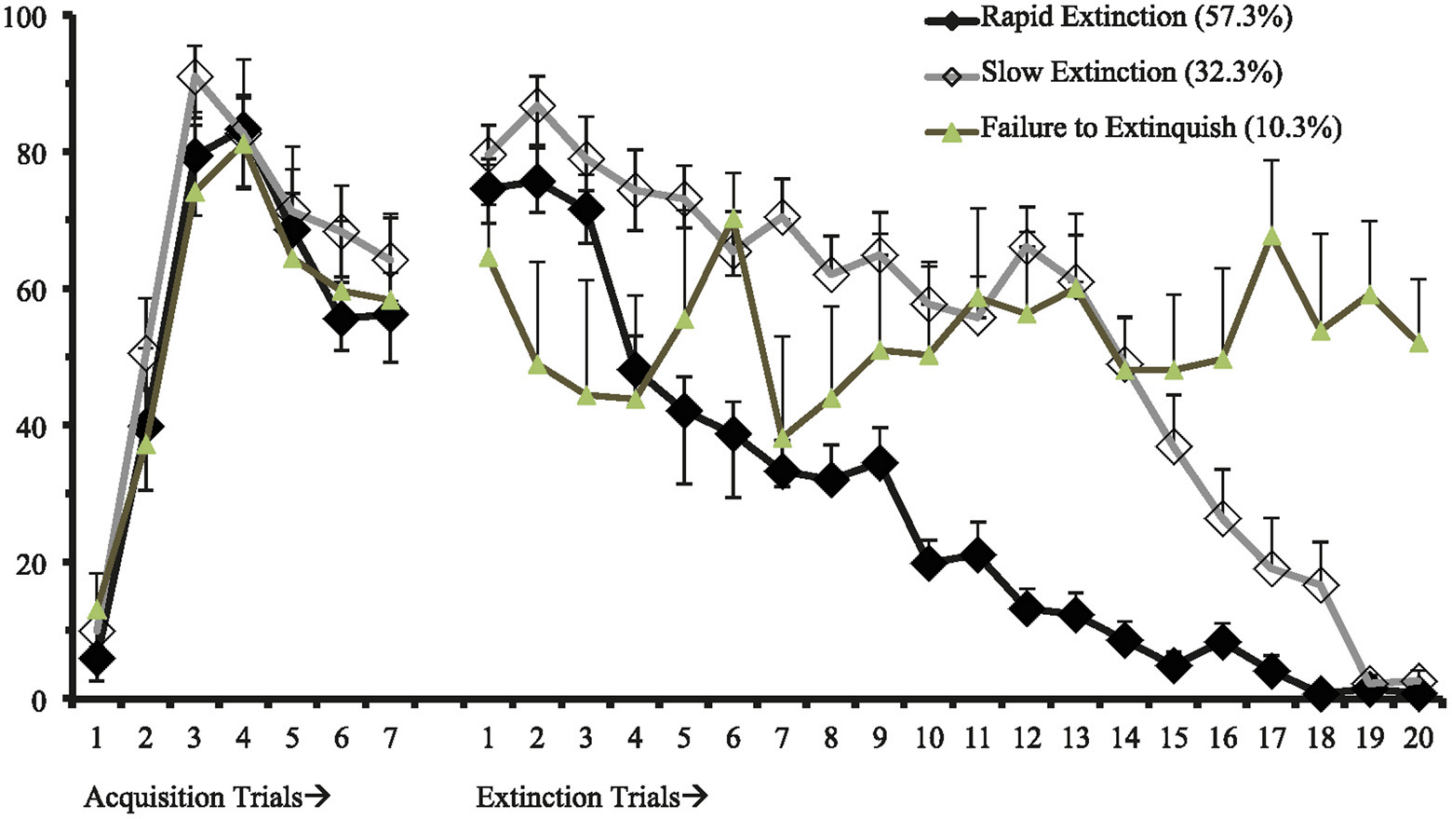
**Three class freezing behavior for 7 fear conditioning and 20 fear extinction trails (*n* = 58)**.

First, we identified a univariate single-class growth model to facilitate model specification for the LCGA with fixed effects on the intercept, slope, and quadratic parameters (Figure 1). This model represents freezing behavior in threat conditioning and extinction assuming a single distribution consistent with typical models of central tendencies. Utilizing the methodology discussed to identify the best fitting model, we found that a 3-class model with linear and quadratic parameters best fit the data (see Table 1; Figure 2). This model demonstrated high posterior probability of correct class specification (entropy = 0.96). The largest class, Rapid Extinction (57.3%), was characterized by a positive linear and a negative quadratic slope for conditioning trials (*EST*_slope_ = 14.06, *SE* = 2.72, *p* < 0.001; *EST*_quadratic_ = −4.19, *SE* = 0.42, *p* < 0.001), an intercept that was significantly distinct from 0 indicating threat conditioning had occurred (*EST*_intercept_ = 73.46, *SE* = 3.10, *p* < 0.001), and a negative slope and positive quadratic for extinction trials indicating a rapid initial rate of decrease (*EST*_slope_ = −6.36, *SE* = 0.63, *p* < 0.001; *EST*_quadratic_ = 0.13, *SE* = 0.03, *p* < 0.001). The second largest class, Slow Extinction (32.3%), was characterized by a positive linear and a negative quadratic slope for conditioning trials (*EST*_slope_ = 18.47, *SE* = 3.49, *p* < 0.001; *EST*_quadratic_ = −4.76, *SE* = 0.54, *p* < 0.001), an intercept that was significantly distinct from 0 indicating threat conditioning had occurred (*EST*_intercept_ = 71.13, *SE* = 3.83, *p* < 0.001), and a positive slope and negative quadratic indicating an initial slow decrease followed by a more rapid rate of decrease during extinction (*EST*_slope_ = 2.03 = SE = 0.79, *p* ≤ 0.01; *EST*_quadratic_ = −0.28, *SE* = 0.03, *p* < 0.001). The smallest class, Failure to Extinguish (10.3%), also demonstrated a positive linear slope and negative quadratic term for conditioning (*EST*_slope_ = 21.31, *SE* = 5.83, *p* < 0.001; *EST*_quadratic_ = −4.67, *SE* = 0.91, *p* < 0.001), and an intercept that was significantly distinct from 0 indicating threat conditioning had occurred (*EST*_intercept_ = 53.57, *SE* = 6.53, *p* < 0.001). However, this class was distinguished from the other classes by a non-significant slope and intercept term for freezing behavior during extinction indicating no change over time relative to the intercept (*EST*_slope_ = −0.29, *SE* = 1.29, *p* = 0.83; *EST*_quadratic_ = 0.02, *SE* = 0.06, *p* = 0.72).

**Table 1:** **Fit indices for 1- to 4-class latent class mixture models of freezing behavior (*n* = 58)**.

**Fit indices**	**AIC**	**BIC**	**SSBIC**	**BLRT**	**Entropy**
**LINEAR WEIGHTS**					
1 Class	15012.96	15074.77	14980.45	–	–
2 Class	14745.79	14815.84	14708.94	*p* < 0.001	0.99
3 Class	14704.81	14783.11	14663.63	*p* < 0.001	0.96
4 Class	14654.61	14741.15	14609.10	*p* = 0.67	0.97
**LINEAR + QUADRATIC WEIGHTS**					
1 Class	14957.96	15023.90	14630.28	–	–
2 Class	14672.00	14750.30	14630.82	*p* < 0.001	0.96
**3 Class**	**14529.38**	**14620.03**	**14481.69**	***p* < 0.001**	**0.96**
4 Class	14556.27	14659.29	14502.08	*p* = 0.09	0.98

*Information Criteria and model fit indices for best fitting model in bold. AIC, Akaike Information Criterion; BIC, Bayesian Information Criterion; SSBIC, Sample Size Adjusted Bayesian Information Criterion; LRT, Lo-Mendell-Rubin Test; BLRT, Bootstrap Likelihood Ratio Test. 1- to 4-class solutions were tested with linear and quadratic parameters*.

### *Post-hoc* analyses

We next conducted a series of ANOVAs to examine mean level differences between the classes at the last time points in conditioning (Conditioning Time 7) and extinction (Extinction Time 20). The ANOVA for class differences in conditioning at Time 7 was non-significant [*F*_(2, 55)_ = 0.92, *p* = 0.40], and additional *post-hoc* analyses using least squares confirmed that there were no significant class differences. The ANOVA for class differences in extinction at time 20 was highly significant [*F*_(2, 55)_ = 92.11, *p* < 0.001]. *Post-hoc* analyses using least squares indicated significant class differences for the Failure to Extinguish class versus the Rapid Extinction class (Mean difference = 49.55, *p* < 0.001) and the Failure to Extinguish class versus the Slow Extinction class (Mean difference = 51.32, *p* < 0.001), but not for the Rapid Extinction class (N.S.) versus the Slow Extinction class (N.S.). These findings indicate that classes did not differ significantly after conditioning, and that at the end of the extinction trails only the Failure to Extinguish class differed significantly from the other two classes.

### Parameters for the identification of extinction classes

Based on this analysis, the parameters for freezing scores during extinction learning for each class were identifiable. These parameters can be utilized and further refined by future studies. Once consistent and accurate parameters are defined in the literature, these populations will be identifiable without needing to utilize the complexity of latent modeling approaches. Rats in the Failure to Extinguish class were distinguishable from the other two classes based on freezing behavior during the final three extinction training trials (average of trials 18–20 ≥ 37.08; see Figure 2). Slow Extinction rats are distinguishable from the other trajectories based on freezing behavior averaged across the middle trials (average of trials 10–15 ≥ 34.67) together with freezing behavior during final trials (average of trials 18–20 ≤ 32.00). The remaining rats are classified as belonging to the Rapid Extinction class. Based on this algorithm, all cases are correctly classified. These parameters should be treated as initial findings to be improved upon with further study.

## Discussion

The current study attempted to identify heterogeneity in defensive behavioral responses to learned threatening stimuli. The goal of this study was to identify clinically meaningful subpopulations in PTC and to demonstrate the efficacy of this approach so as to better inform translational models of threat response in humans. By using a PTC paradigm, heterogeneous patterns of freezing behavior in response to the CS/US pairing (conditioning) followed by the CS presented alone (extinction) were identified. Based on evidence from the literature on the longitudinal course of stress responses in humans (Bonanno et al., 2008, 2012a,b; Deroon-Cassini et al., 2010; Galatzer-Levy et al., 2010, 2011b, 2012; Galatzer-Levy and Bonanno, 2012), along with evidence from the animal literature that rodent freezing behavior is highly non-normal during extinction learning even when exposed to identical experimental conditions (Cavigelli and McClintock, 2003; Cavigelli et al., 2006; Sotres-Bayon et al., 2008; McEwen et al., 2012), we hypothesized that the unexplained variability in animal models of Pavlovian defensive learning could be captured by multiple, latent, heterogeneous patterns of response. To test this hypothesis, a cohort of adult male outbred rats (*n* = 58) (Bush et al., 2007) were examined for heterogeneity in threat conditioning and extinction learning using LCGA, a data analytic approach for the identification of latent heterogeneous longitudinal trajectories (Muthen, 2004). A piecewise model (Flora, 2008) was used to disentangle differences among classes on both threat conditioning and threat extinction learning.

A three class solution best fit the data. The majority class, *Rapid Extinction* (57.3%), extinguished rapidly and completely as evidenced by a significant negative quadratic parameter suggestive of a rapid decrease in freezing behavior during extinction. The second largest class, *Slow Extinction* (32.3%), also reached basal levels of freezing behavior through extinction training but at a slower pace, suggested by a significant positive quadratic parameter. Finally, we observed a *Failure to Extinguish* (10.3%) class of rats. This class failed to acquire extinction learning. The classes were not significantly different in freezing behavior during conditioning, indicating that initial threat-elicited defensive response is more or less uniform across these groups, and that differences manifest primarily in the ability to learn that the CS no longer signals harm. These results suggest that conditioning and extinction learning are distinct but related learning processes. This indicates that mechanisms underlying the extinction and not conditioning may explain patterns of adaptation. These trajectories differed somewhat from those previously identified with this data (Bush et al., 2007). However, the model that included the Failure to Extinguish class demonstrated stronger model fit and is more ecologically valid.

It is important to acknowledge that a more definitive claim about common patterns or phenotypic responses in conditioning will require replication. The impact of these findings would be greatly increased if similar patterns and proportions were observed in an independent population of rodents exposed to the same conditioning paradigm. Secondly, the data analytic methods utilized require larger samples then typically used in this kind of research, making further exploration somewhat prohibitive. Further, the current study did not attempt to identify mechanisms and it remains unclear if the observed heterogeneity is due to genetic, epigenetic, or uoncontrolled environmental conditions. If the observed heterogeneity is due to environmental conditions, those conditions are likely subtle, as the experiment was uniform and closely controlled.

Despite these limitations, the current study has important implications for the understanding of heterogeneity in responses to stressors. Evidence for heterogeneous stress responses in experimental Pavlovian defensive learning may be important to the study of distress- and stress-related psychopathology in humans. The development of such translational models may lead to important discoveries regarding environmental and biological mechanisms and correlates of chronic stress and resilience. To achieve this goal, it is important to utilize modeling techniques that are appropriate both for identifying heterogeneity in the course of behavioral responses and for testing hypotheses related to those differences.

While the current study focused on defensive learning, there are other adaptive functions that are achieved by forming associations between biologically driven responses and previously neutral stimuli (Cardinal et al., 2002; LeDoux, 2012). Researchers are commonly interested in knowing why some adapt well to novel stimuli while others do poorly. Why do some develop significant and prolonged stress reactions following exposure to a potentially traumatic event while others cope well? Why do some develop addictions when exposed to a potentially addictive substance while others do not? Why do some eat in excess leading to obesity when they are given free access to food while others do not? All of these questions are examined through translational animal models in an attempt to understand complex biological processes that explain meaningful differences between groups that behave distinctly. Elucidation of individual differences that capture distinct populations is important for identifying individual differences in less easily observable neurobiological processes. Attending to meaningful heterogeneity can inform the understanding of any contexts where learned triggers activate biologically driven behavioral responses.

### Conflict of interest statement

The authors declare that the research was conducted in the absence of any commercial or financial relationships that could be construed as a potential conflict of interest.
